# Reversible hydrogen storage in reactive hydride composites under 400 K

**DOI:** 10.1038/s41467-026-75313-0

**Published:** 2026-07-10

**Authors:** Nicholas J. Hall, David M. Grant, Jacob L. Prosser, Jamie E. Ramshaw, Muhammad Saad Salman, Luke J. Woodliffe, Sweta Munshi, Guillaume Esser, Sanliang Ling, Marek Polanski, Yaroslav Filinchuk, Hujun Cao, Ping Chen, Martin Dornheim

**Affiliations:** 1 Advanced Materials Research Group, Faculty of Engineering, University of Nottingham, Nottingham, United Kingdom; 2 Institute of Condensed Matter and Nanosciences, Université Catholique de Louvain, Louvain-la-Neuve, Belgium; 3 Department of Functional Materials and Hydrogen Technology, Military University of Technology, Warsaw, Poland; 4 Dalian Institute of Chemical Physics, Chinese Academy of Sciences, Dalian, China

**Keywords:** Hydrogen storage materials, Materials chemistry

## Abstract

Hydrogen storage remains a key challenge for widescale adoption of hydrogen as an energy vector. Lightweight complex hydrides offer high storage densities but suffer from hydrogen release/cyclability above the temperatures required for practical use. Here, we report on discoveries in ternary Reactive Hydride Composites (RHCs). We systematically tuned the LiBH₄ content in the well-established Mg(NH₂)₂ - LiH framework, achieving reversible hydrogen release at temperatures starting below 393 K and a capacity of 3.1 wt%; a decrease of 100 K compared to the Mg(NH₂)₂ - LiH system.This is a crucial step towards the use of complex hydride-based hydrogen carriers for stationary and onboard hydrogen storage applications. We demonstrate a reversible RHC within the utilisation range of low-grade waste heat from a fuel cell, alongside offering insight into the reaction pathways in these RHCs to inform the design of future materials.

## Abstract (web summary)

Lightweight complex hydrides offer high storage densities but need high release temperatures. The work presented here details a reactive hydride composite system tuning LiBH₄ in Mg(NH₂)₂–LiH to enable a 3.1 wt% reversible H₂ release below 400 K.

## Introduction

The transition to a low-carbon economy hinges on scalable solutions for clean energy storage and distribution, particularly in sectors where direct electrification is impractical. Hydrogen (H_2_) holds significant potential as a clean energy vector due to its high energy content (120 mJ kg^−1^^1^) and clean combustion, offering pathways to decarbonise transport, industry, and power generation. Its use in fuel cells^2^ and internal combustion engines^3^ makes hydrogen a flexible alternative to fossil fuels. However, the deployment of hydrogen technologies is fundamentally constrained by storage and delivery challenges.

The major challenge with utilising hydrogen as an energy vector is the inherently low volumetric energy density at ambient conditions^4^. To address the issue of low volumetric energy density, compressed gas (typically 350–700 bar) or cryogenic liquid hydrogen are currently the most viable solutions. However, these solutions impose energy penalties through an increase in system weight, encounter low efficiencies due to compression/liquefication and raise safety concerns^5^. At the material level solid-state hydrogen storage offers an appealing alternative by increasing volumetric energy density beyond that of cryogenic and compressed gas approaches without the need for high pressures (1000 bar) or cryo-temperatures^6^. Metal hydrides have been extensively studied as potential solid-state hydrogen storage, however poor gravimetric capacities for hydride systems, such as 1.4 wt % for LaNi_5_^6,7^, have limited their practical use. To compete with battery technologies, which typically achieve system level energy densities of ca. 0.2 kWh/kg^8^, consideration of the whole system (hydride tank, heat management components, and fuel cell) is needed.

A simple calculation demonstrates that a car (90–150 kW) requiring 5 kg of hydrogen with a fuel cell weighing 250 kg, operating at 60% efficiency^9^, supplied with 5 kg of hydrogen from a hydride of 3 wt% contained in a hydrogen storage tank (HS) where two-thirds of the mass of the tank is structural due to high pressure and heat management components, yields a system energy density of ca. 0.2 kWh/kg comparable with batteries. Allowing for future fuel cells (FCs) weight reduction to 150 kg only increases this to a 0.25 kWh/kg system. Thus, to make a step change, hydrides must operate at near ambient temperatures and utilise the waste heat from an FC, which is in the range of 60–80 °C^10,11^. For an FC + HST to be competitive and exceed future battery targets, they would require the reversible capacity of the materials to be closer to 4 wt% combined with light weighting of HST due to the low pressures. Assuming 50% of tank mass is hydride this results in a system energy density for FC + HS of >0.4 kWh/kg with all the advantages of not having to compress hydrogen gas to 350 or 700 bar. This step change has been the challenge for over 50 years to deliver hydrogen from solid state storage at sufficient energy densities at low pressure and ambient temperatures for land transport. Here in this paper, we believe we have made a significant breakthrough based on a reactive hydride composite with unusually high ratios of LiBH_4_.

Complex hydrides, including alanates (e.g., NaAlH₄^12^) and borohydrides (e.g., LiBH₄^13^) offer higher hydrogen capacities than intermetallic hydrides or physisorption materials^10^, whilst also offering tuneable thermodynamic properties. However, their practical use is hindered by thermodynamic and kinetic limitations. For instance, LiBH₄ can theoretically desorb 18.5 wt%, however only 13.9 wt% hydrogen release is observed experimentally starting at 573 K, requiring temperatures as high as 873 K to reach just 9 wt% capacity^14^. The reversibility of LiBH_4_ is equally challenging, needing 873 K and 350 bar of hydrogen for rehydriding^15,16^. Full reversibility is hindered by two primary mechanisms: the segregation of boron into stable compounds (such as Li_2_B_12_H_12_) and its subsequent loss through volatile species like diborane (B_2_H_6_). Other promising materials, such as Mg(NH₂)₂ and LiH, have similarly high-temperature desorption profiles or produce undesirable byproducts such as ammonia (NH_3_)^17–19^.

These limitations have prompted extensive research over the last 20 years into strategies for modifying the hydrogen release and uptake behaviour of complex hydrides. Approaches such as nanoscaling^20^, catalyst doping^15,21–23^, and compositional tuning have yielded promising improvements, yet often with trade-offs in capacity or stability through cycling^19^. Among these efforts, the formation of multicomponent systems, known as reactive hydride composites (RHCs), have emerged as a powerful route to circumvent intrinsic kinetic and thermodynamic barriers while leveraging the favourable storage potential of complex hydrides. RHCs combine multiple hydrogen-containing phases that undergo cooperative reactions, lowering dehydrogenation enthalpies and enhancing reversibility^24–26^. The RHC approach trades some theoretical hydrogen capacity for improved kinetics, system cyclability, and practical viability through theoretical operating temperatures closer to the operating temperature of a fuel cell^27,28^.

Binary RHC systems such as MgH_2_–LiBH_4_ (ca. 9.8 wt % H_2_ release)^22,29–32^, Mg_2_NiH_4_–MgH_2_ (ca. 4.8 wt % H_2_ release)^33^, Mg(NH_2_)_2_–LiH (ca. 5.6 wt % H_2_ release)^34–40^, and Mg(NH_2_)_2_–LiBH_4_ (ca. 11 wt % H_2_ release)^41^ have been investigated for their favourable hydrogen storage properties. Among these, the Mg–Li–N–H system in a 1:2 molar ratio (Mg(NH₂)₂:LiH) stands out due to its low onset dehydrogenation temperature (413 K) and moderate theoretical reaction enthalpy of ~40 kJ mol⁻¹ H₂^42^. The system proceeds via a solid-state reaction forming the mixed-metal imide Li₂Mg(NH)₂ (Eq. (1)):

1 $${Mg}{\left(N{H}_{2}\right)}_{2}+2{LiH}\rightleftharpoons L{i}_{2}{Mg}{\left({NH}\right)}_{2}+2{H}_{2}$$

This solid-state reaction enables reversible hydrogen storage over multiple cycles^43^, although there is release of NH_3_ from side reactions, which lead to degradation of capacity over time^44^. The equilibrium-based reaction pathway promotes faster hydrogen release and uptake compared to systems reliant on single-component phase transitions, while also mitigating the kinetic limitations often associated with complex hydrides.

Furthermore, the ability to pre-synthesise the ternary imide Li₂Mg(NH)₂ has facilitated mechanistic studies and kinetic optimisation, revealing opportunities to tailor reaction rates and cycling behaviour as shown in recent work by Cao et al.^42^, which explored synthesis of the 1:2 system from magnesium waste.

Despite the favourable thermodynamics, the practical application of systems such as this has been constrained by sluggish kinetics. Specifically, the Mg(NH_2_)_2_–LiH system has been shown in numerous studies to exhibit a relatively high activation energy for hydrogen desorption of 127 kJ mol⁻¹^34,43,45,46^, which significantly limits hydrogen release at moderate temperatures (below 373 K).

To address this, researchers have explored modifying the Mg–Li–N–H system by incorporating borohydrides such as LiBH₄. Hu et al. demonstrated that small additions of LiBH₄ (≤0.3 molar equivalents) weaken N–H bonds via in-situ chemical interactions, reducing activation barriers and enhancing sorption rates^47^. Similarly, Zhang et al. reported that incorporating a small amount of Mg(BH₄)₂ and LiBH₄ into the Mg(NH₂)₂–LiH system alters the reaction pathway. Interestingly, this addition highlighted that the 6-9 ratio of Mg(NH_2_)_2_–LiH was favoured over the 1:2 ratio, resulting in the formation of Li₂Mg₂(NH)₃ and LiNH₂^48^ (Eq. (2)):

2 $$6{Mg}{\left(N{H}_{2}\right)}_{2}+9{LiH}\rightleftharpoons 3{LiN}{H}_{2}+	 3L{i}_{2}M{g}_{2}{\left({NH}\right)}_{3}+9{H}_{2} \,\,\, {{\mbox{or}}} \,\,\,\, 2{Mg}{\left(N{H}_{2}\right)}_{2} \\ 	+3{LiH}\rightleftharpoons {LiN}{H}_{2}+L{i}_{2}M{g}_{2}{\left({NH}\right)}_{3}+3{H}_{2}$$

These findings highlight the sensitivity of complex hydride systems to stoichiometric and compositional changes, suggesting that tuning the LiBH₄ content could further optimise both thermodynamics and kinetics.

Recent studies have shown that increasing LiBH₄ concentrations in Mg(NH₂)₂–LiH–LiBH₄ composites significantly accelerates hydrogen uptake and release. Among the studied *y*Mg(NH_2_)_2_-*z*LiH-*x*LiBH_4_ systems are the 6-9-1 and 6-9-12 composites, which demonstrate rapid dehydrogenation, completing hydrogen release within ~10 min at temperatures near 453 K^24,25,49^. For the 6-9-1 composition, a proposed reaction mechanism has been outlined by Zhang et al.^48^, indicating that LiBH₄ not only participates in hydrogen release but also plays a role in the formation of stable products (Eq. (3)):

3 $$6{Mg}{\left(N{H}_{2}\right)}_{2}+9{LiH}+{LiB}{H}_{4}\rightleftharpoons L{i}_{4}\left(B{H}_{4}\right){\left(N{H}_{2}\right)}_{3}+3L{i}_{2}M{g}_{2}{\left({NH}\right)}_{3}+9{H}_{2}$$

Notably, the 6-9-1 system begins to desorb hydrogen at 447 K, while the 6-9-12 variant exhibits limited hydrogen release from temperatures as low as 363 K^24^. The latter system achieves a dehydrogenation enthalpy of just 24 kJ mol⁻¹ H₂, well within the 2025 U.S. DOE target range for onboard hydrogen storage applications^50^. Despite these improvements, kinetic barriers still limit the full exploitation of their thermodynamic potential, with reasonably fast (<10 min) hydrogen release rates below 100 °C remaining unattainable under practical conditions^24^. These results illustrate both the progress and ongoing challenges in engineering RHC systems that simultaneously meet thermodynamic and kinetic performance benchmarks.

In this study, we investigate the influence of unusually high ratios of LiBH₄ on dehydrogenation in the 6Mg(NH_2_)_2_−9LiH-*x*LiBH_4_ systems (*x* = 1, 6, 12, 18, 24); in particular focussing on compositions and reaction pathways with high ratios of LiBH₄ which form complex structures through reversible pathways. Using a combination of thermogravimetric analysis, differential scanning calorimetry, mass spectrometry and temperature-ramped Synchrotron Powder X-ray Diffraction, we identify partial melt-like behaviour that appears to play a critical role in hydrogen release. Additionally, increased LiBH_4_ content appears to hinder NH₃ release, addressing a key challenge in N-containing hydride systems. Notably, our findings demonstrate that the 6-9-18 system can reversibly desorb 3.1 wt% H₂ from 350 K, a significant improvement over previously reported RHCs, in the temperature range of practical waste heat from a fuel cell. These insights provide perspectives on phase evolution and reaction mechanisms in RHCs, highlighting the potential for further performance enhancements, to reach the gravimetric target of 4 wt% hydrogen.

## Results

### Hydrogen storage properties

**Fig. 1:**
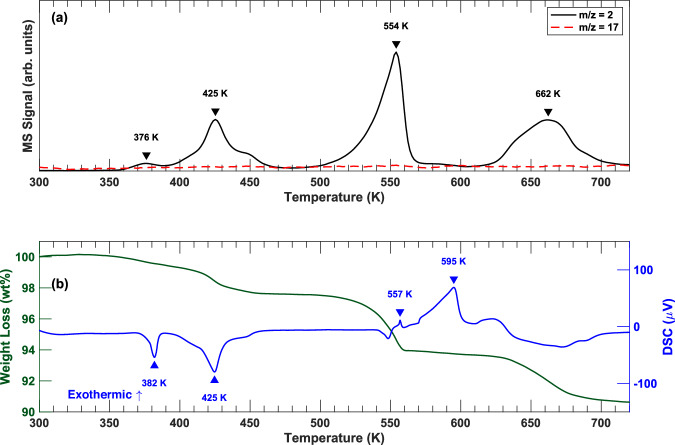
TGA-DSC-MS combined plot for the 6-9-18 sample. The data illustrates the complex dehydrogenation reactions of the 6Mg(NH_2_)_2_-9LiH-18LiBH_4_ RHC between 298 K and 723 K at a ramp rate of 2 K min^−1^. **a** The MS signal for m/2 = 2 and M/Z = 17 over the dehydrogenation, no NH_3_ is detected above background levels in this sample. **b** Combined TGA-DSC data showing three distinct weight loss zones, 298–475 K, 500–560 K and 630–700 K.

Thermal analysis of the 6-9-*x* systems (*x* = 1, 6, 12, 18, 24) was performed using TGA-DSC-MS under argon flow. All samples exhibited multiple hydrogen release steps (Fig. 1). For the 6-9-18 system, the first hydrogen release occurred at 350 K. All samples except the 6-9-1 showed a major hydrogen release and corresponding mass loss below 450 K, with the 6-9-18 system showing major hydrogen release at 422 K. Two additional major mass loss events were observed at 500 K and 625 K across the samples, corresponding to further hydrogen release. These later events likely involve the decomposition of excess LiBH₄ and formation of thermodynamically stable phases such as Mg_3_N_2_ and LiMgN (Supporting Information S8), as well as probable B_2_H_6_ release.

**Fig. 2:**
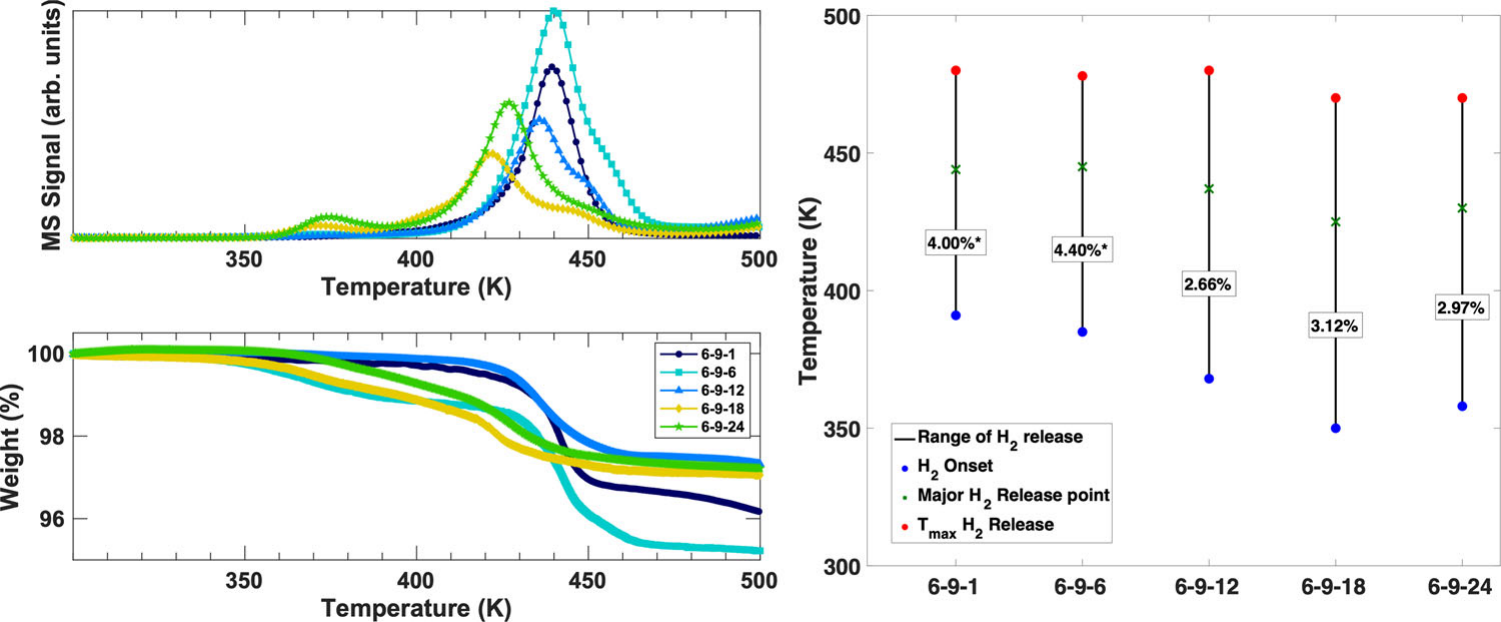
TGA-MS data highlighting the changes in hydrogen release across the 6-9-x samples. (Left) TGA-MS data, highlighting the difference in reaction of the 6-9-18 and 6-9-24 samples against the previously studied 6-9-1,6-9-6 and 6-9-12 systems. The H_2_ signal from the MS (m/z = 2) has arbitrary height due to differing sample masses on the TGA (Right) A graphic visualisation of the TGA-MS data illustrating gravimetric capacities (TG), detectable H_2_ onset temperatures (MS), major release point and temperature range. Major H_2_ release is calculated from the derivative of the TG data, highlighting the area of steepest weight loss. * Indicates kinetic challenges in these systems from the literature.

Within the operationally relevant temperature range of 298–500 K, the systems exhibited gravimetric capacities between 2.66 wt% (6-9-12) and 4.40 wt% (6-9-6), aligning with values reported in prior studies^24,25^. Notably, the 6-9-18 and 6-9-24 systems achieved higher capacities (3.12 and 2.97 wt%, respectively) compared to the 6-9-12 (2.66 wt%), despite the increased LiBH₄ content. Figure 2 shows that for the 6-9-6 and 6-9-18 samples, minor mass losses occur below 350 K. However, detectable hydrogen evolution begins from 350 K in the 6-9-18 system and from 385 K in the 6-9-6 system.

Compared to previous literature at an identical heating rate, the 6-9-1 system showed the peak hydrogen release at 444 K, slightly lower than the 453 K reported by Gizer et al.^25^. In the 6-9-12 system, the peak H_2_ release occurred at 437 K, similar to prior reports of 440 K^24^, with the initial H_2_ release at 368 K again close to the 363 K previously reported. Figure 2 highlights the fact that both the 6-9-18 and 6-9-24 compositions exhibit measurable H₂ evolution, beginning at 350 K and 358 K, respectively. Mass loss in the TGA is observed from ~348 K in the 6-9-6 and 6-9-18 samples. No NH_3_ release was detected by MS within the 298–500 K range for the 6-9-12, 6-9-18, or 6-9-24 systems, while weak NH_3_ signals were observed in the 6-9-1 and 6-9-6 samples, which may result in the larger gravimetric capacities.

**Fig. 3:**
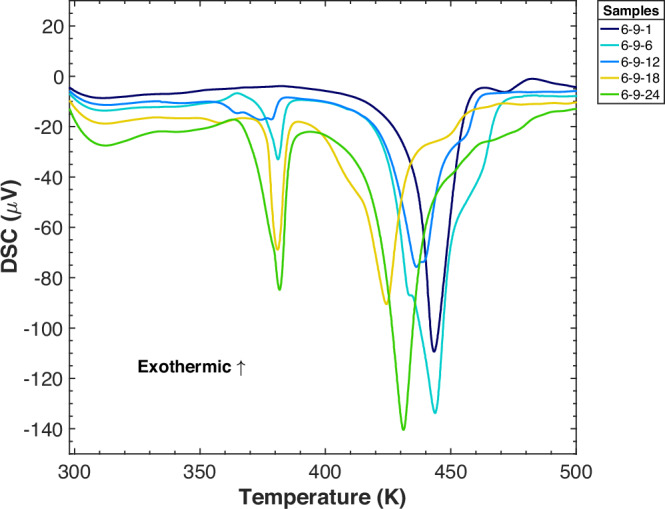
DSC data of the 6-9-x (x = 1, 6, 12, 18, 24) systems. Data collected during dehydrogenation from 298 K to 500 K at 2 K min^−1^.

The DSC data is indicative of the complexity of the reactions occurring between 298 K and 500 K, with multiple reactions present and clear disparities between the systems (Fig. 3). All systems except 6-9-1 displayed a first endothermic event near 380 K, typically assigned to the LiBH_4_ orthorhombic to hexagonal phase transition. The 6-9-1 in the measurements here is in line with prior experiments by Gizer et al. where the phase transition is unobserved^25^, and this is unexplained. For the 6-9-18 and 6-9-12 systems enthalpies calculated from peak integration were 74.2 J g⁻¹ and 35.8 J g⁻¹, respectively. These values were substantially higher than predicted for the LiBH₄ phase transition alone. In the 6-9-12 system, the first endothermic event appeared to consist of multiple overlapping features, previously unseen. The second endothermic peak, corresponding to the main hydrogen release, occurred at 422 K for the 6-9-18 system and at 437 K for the 6-9-12 system.

### Structural evolution through dehydrogenation

**Fig. 4:**
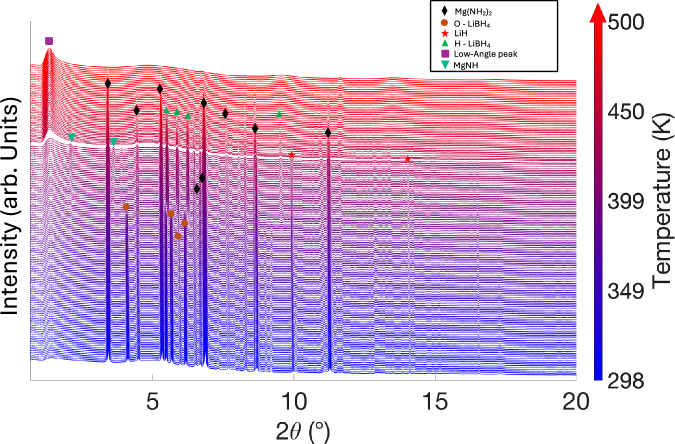
T-ramp SR-PXRD from 298 K to 500 K of the 6-9-18 system. The major peaks are indexed including a low angle peak at 1.3° 2θ, and two LiBH_4_ phases: the room temperature orthorombic (O - LiBH_4_), as well as the hexagonal (H - LiBH_4_) which appears from ca. 384 K.

**Fig. 5:**
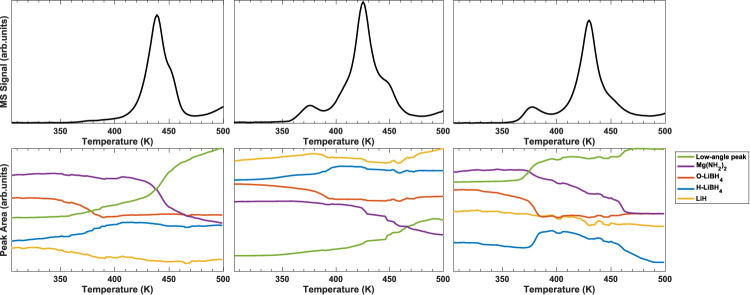
Peak areas compared with H_2_ (M/Z = 2) signal intensity from the MS data, utilised for understanding phase composition, in the 6-9-12 (left), 6-9-18 (middle) and 6-9-24 (right) systems. Low-angle peak refers to the unknown peak appearing at 1.3° 2θ in the SR-PXRD measurements.

**Fig. 6:**
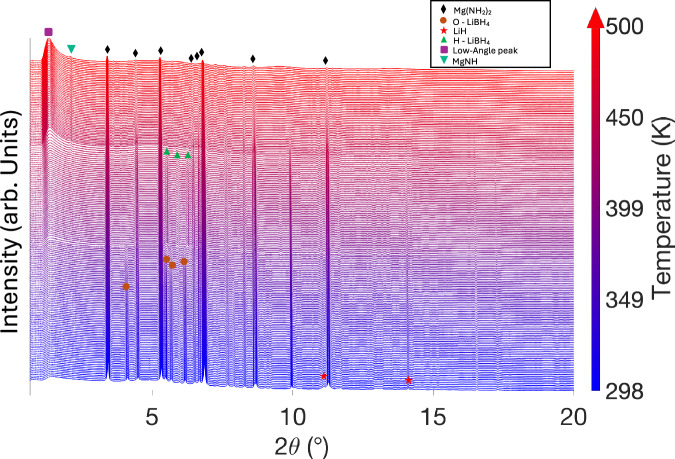
T-ramped SR-PXRD from 298 K to 500 K of the 6-9-12 system. The major peaks are indexed, including a low angle peak at 1.3° 2θ and two LiBH_4_ phases: the room temperature orthorombic (O - LiBH_4_), as well as the hexagonal (H - LiBH_4_) which appears from ca. 384 K.

Temperature-ramped synchrotron powder X-ray diffraction (SR-PXRD) revealed significant structural evolution in all systems (Figs. 4–6). In the 6-9-1 system, diffraction peaks corresponding to Li₂Mg₂(NH)₃ and Li₄(BH₄)(NH₂)₃ formed during heating at 450 K, consistent with prior studies^25^.

For the 6-9-6, 6-9-12, 6-9-18, and 6-9-24 systems, a broad low-angle peak at  1.3° 2θ was observed (Fig. 4). This peak was present even at room temperature after milling the composites before dehydrogenation, albeit weakly, and intensified with heating, correlating with hydrogen release. In the 6-9-6 system, this peak initially grew but diminished at higher temperatures as more stable crystalline products formed. In the 6-9-18 and 6-9-24 systems, the low-angle peak persisted at elevated temperatures.

At 500 K, diffraction patterns from the 6-9-18 and 6-9-24 systems showed broad signals and minimal crystalline reflections apart from the low-angle peak (at 1.3° 2θ) and weak Pm3̅m reflections corresponding to a cubic phase with a lattice parameter of ~3.02 Å. The broad signal is across the LiBH_4_ peaks from 420 K to 500 K, suggesting the formation of nanocrystalline, molten or amorphous products at high temperatures.

The LiBH₄ orthorhombic-to-hexagonal phase transformation was observed at ~390 K in all systems containing LiBH₄, consistent with previous studies^22,23,25,51^. However, aside from this transition, in the *x* = 12, 18 and 24 systems limited additional structural phases were detected in the diffraction patterns, suggesting the formation of amorphous, molten or poorly crystalline products as the LiBH_4_ content increases.

Phase composition analysis showed that in the 6-9-12 system, Mg(NH₂)₂ and LiH reacted between 340–430 K with minimal hydrogen evolution. Above 440 K, Mg(NH₂)₂ appears to react with LiBH₄, leading to significant hydrogen release (Fig. 5). MgNH formation, a stable intermediate previously found in the decomposition of Mg(NH_2_)_2_ and in prior 6-9-12 characterisation^17,24^, began at 440 K and persisted throughout the thermal ramp, alongside the continued growth of the 1.3° 2θ peak. Weak diffraction peaks consistent with a LiNH₂–LiBH₄ compound were observed^24^, but no crystalline Li₄(BH₄)(NH₂)₃ was detected under the current experimental conditions, unlike the 6-9-1 and 6-9-6 systems.

In the 6-9-18 system, LiH consumption began from ~330 K, with MgNH forming between 360 and 430 K but disappearing above 450 K (Fig. 5). The transition from Mg(NH₂)₂–LiH to Mg(NH₂)₂- LiBH₄ reaction pathways occurred at ~430 K. In the 6-9-24 system, Mg(NH₂)₂ and LiBH₄ reacted from 380 K, while LiH decreased more gradually. No MgNH formation was observed in the 6-9-24 system.

The low-angle peak at 1.3° 2θ (d-spacing = 15.63 Å) was consistently observed in the 6-9-6, 6-9-12, 6-9-18, and 6-9-24 compositions. There are no other reflections observed in the data for this phase, suggesting that this peak corresponds to a large superstructure with poor ordering. The growth of the low-angle peak in the SR-PXRD is correlated with hydrogen release and a decrease in LiBH_4_ content (shown in Fig. 5), indicating a partially decomposed Li–Mg–N–B–H intermediate which is either a layered or intercalated structure. Other possible explanations include nanoscale or amorphous phases, although the sharpness of the peak makes these less likely options, but they cannot be excluded totally. The low angle peak appears and then reduces as Li_2_Mg_2_(NH)_3_ forms in the 6-9-6 composite and was also observed at the same d-spacing with other capillary materials during preliminary measurements at UCLouvain (Supporting Information S2).

Alongside the low angle peak there are weak peaks relating to a primitive cubic cell (${{\rm{Pm}}}\bar{3}{{\rm{m}}}$) with a lattice parameter of 3.02 Å (indexed in Table 1) observed weakly from 450 K onwards in the 6-9-18 system. The peaks are seemingly separate to the low angle peak observed at 1.3°, and the peaks for this phase are very weak (Supporting Information S7) and are most prominent in the 6-9-18 system at 500 K, however these peaks can also be weakly observed in the 6-9-12 and 6-9-24 samples at 500 K.

**Table 1:** Indexed PXRD pattern for the weakly observed primitive cell (${{\rm{Pm}}}\bar{3}{{\rm{m}}}$) at 500 K, compared to a ${{\rm{Pm}}}\bar{3}{{\rm{m}}}$ cell with a lattice parameter of 3.02 Å

2θ_obs_ (°)	H K L	2θ_calc_ (°)	2θ_obs_-2θ_calc_ (°)	d_obs_ (Å)	d_calc_ (Å)
6.73	001	6.73	–0.007	3.02	3.02
9.52	011	9.53	–0.009	2.14	2.14
11.67	111	11.68	–0.011	1.75	1.74
13.48	002	13.49	–0.013	1.51	1.51
15.08	012	15.09	–0.014	1.35	1.35
19.11	202	19.12	-0.018	1.07	1.07
21.39	301	21.41	–0.020	0.96	0.96
23.46	222	23.48	–0.022	0.87	0.87

## Discussion

Here we report a step change in lowering the onset temperature of H_2_ release by over 100 K compared to the original 6Mg(NH_2_)_2_–9 LiH system without LiBH_4_, accompanied by a corresponding different reaction pathway for the 6-9-18 and 6-9-24 systems, offering potential for improved cycling stability due to the lack of NH_3_ release. The thermal and structural analysis presented here reveals insights into the first cycle dehydrogenation of Mg(NH₂)₂–LiH–LiBH₄ RHCs and their further optimisation potential. In addition, to verify reversibility Pressure Composition Isotherm measurements and thermal characterisation was conducted over 3 cycles (Supporting Information S6).

The 6-9-1 and 6-9-6 systems, despite their higher capacities, are not fully reversible. The 6-9-1, for example, has been shown to reduce to 96% capacity after 14 cycles, as well as showing a significant reduction in the kinetics by a factor of ca. 2.6 for the dehydrogenation reaction^44^. Gizer et al. demonstrated the cycling of 6-9-1 up to 20 times with a negligible drop in capacity, but they did note a similar reduction in the kinetics of the 6-9-1 system over the cycling^25^. The detection of NH₃ release above 473 K in the 6-9–1 composite, with a temperature 30 K above the cycling temperature used by Gizer et al.^25^, points to NH_3_ evolution as a likely contributor to the capacity degradation observed during cycling.

For the 6-9-6 system, the first dehydrogenation cycle contains a loss of ca. 1 wt%, with no observable release of NH_3_ or H_2_. The cause for this weight loss is not clear. It cannot at present be excluded that in this sub-stoichiometric RHC boron related species such as diborane (B_2_H_6_) or borane (BH_3_) are released as potential decomposition products of LiBH_4_. Prior studies have shown that during the dehydrogenation of less stable borohydrides, desorbing at lower temperatures (below 473 K) release B_2_H_6_, whereas more stable borohydrides that release at over 523 K preferentially release H_2_^52–54^. This is due to B_2_H_6_ decomposing at ca. 523 K^55^. Both theoretical^56^ and experimental studies^57^ have shown previously that species like B_2_H_6_ can be formed in the dehydrogenation reactions of LiBH_4_, although B_2_H_6_ is difficult to observe experimentally with mass spectrometry, requiring Raman spectroscopy^57^.

Further evidence of boron species impacting reaction pathways is clear in the 6-9-6 system, where the in-situ SR-PXRD highlights distinct difference to the 6-9-1 system. At high temperatures after dehydrogenation, the 6-9-1 has two strong crystalline phases present, Li_2_Mg_2_(NH)_3_ and Li_4_(BH_4_)(NH_2_)_3_. In the 6-9-6, only the Li_2_Mg_2_(NH)_3_ phase is observed to be crystalline, with no evidence of the Li_4_(BH_4_)(NH_2_)_3_ phase or any other crystalline B-containing phase forming. This could be explained by preferential partial B_2_H_6_ or borane release earlier in the reaction mechanism or the formation of amorphous boron rather than forming Li_4_(BH_4_)(NH_2_)_3_^57^.

The 6-9-12 has been shown to have improved reversibility against the 6-9-1 and 6-9-6 systems^24,49^, with a measured capacity of 2.66 wt% (Fig. 2), consistent with the literature^24,49^. In the TGA data for the 6-9-12, there is no significant mass loss below 400 K, much like the 6-9-1 (Fig. 2), although it has an earlier onset of hydrogen release at 368 K compared to 391 K the 6-9-1. Studies on the 6-9-12 however have shown release at 371 K over more than 140 h^24^, implying that kinetic limitations at lower temperatures prevent this system from ambient operation.

Here we report two systems, the 6-9-18 and 6-9-24, which both display significantly lower hydrogen desorption onset temperatures compared to previous 6-9-*x* composites, releasing H_2_ from 350 K and 358 K respectively: ca. 100 K lower than the reported values of 458 K for the Mg(NH_2_)_2_–LiH system without LiBH_4_^58^. In these systems the early hydrogen release (below 400 K) leads to a fast hydrogen release of 1.2 wt% for the 6-9-18 and 1 wt% for the 6-9-24, compared to 0.2 wt% for the 6-9-12, Fig. 2. The hydrogen MS signal is also much stronger below 400 K, illustrating a difference in reaction pathway from the 6-9-12 to the 6-9-18 and 6-9-24 samples, and further evidencing the key role of LiBH₄ concentration in modulating the dehydrogenation onset. Table 2 summarise the key observations for each of the systems. Initial testing (Supporting Information S6) shows the 6-9−18 continues to be a reversible system when tested under PCI conditions, with a plateau of around 100 bar at 403 K. After 3 cycles a 6-9-18 sample was tested via TGA-DSC-MS and demonstrated a 3 wt% capacity but kinetics were noticeably slower, requiring a slower heating rate of 1 K min^−1^ and H_2_ onset shifting to 388 K. Further work into the kinetics and stability of this system is required to investigate if there is any reduction in capacity over multiple cycles (>50), for practical applications.

**Table 2:** Phases observed via in-situ SR-PXRD of the 6-9-x systems

	$L{i}_{2}M{g}_{2}{\left({NH}\right)}_{3}$	${{Li}}_{4}\left(B{H}_{4}\right){\left(N{H}_{2}\right)}_{3}$	${MgNH}$	1.3° Peak	${{\rm{Pm}}}\bar{3}{{\rm{m}}}$
6-9-1	Yes ca. 445 K.	Yes ca. 445 K	No	No	No
6-9-6	Yes ca. 450 K	No	No	Yes ca. 350 K, disappears ca. 450 K	No
6-9-12	No	No	Yes ca. 400 K	Yes ca. 350 K	Yes, ca. 420 K
6-9-18	No	No	Yes ca. 350 K, disappears ca. 420 K	Yes ca. 350 K	Yes, ca. 420 K
6-9-24	No	No	No	Yes ca. 350 K	Yes, ca. 420 K

The multiple hydrogen release steps observed by the DSC and MS data (Figs. 2, 3) along with products in Table 2 provides further evidence of the presence of distinct reaction pathways that are composition dependent. The large enthalpies, such as 74.2 J g^−1^ for the first endothermic event in the 6-9-18 system, exceeds the expected value for the LiBH₄ phase transition^51^. This is seen across all the 6-9-*x* samples implying that additional reactions are occurring in this temperature range. The in-situ SR-PXRD data supports the idea of additional reactions, with the emergence of a low-angle diffraction peak unaccounted for by known phase transitions. This feature likely corresponds to an intermediate, facilitating the low-temperature hydrogen release and potentially suppressing NH₃ evolution, a persistent challenge in nitrogen-based hydrides.

The 6-9-1 system, as mentioned above, follows a previously reported reaction pathway^25^ forming Li₂Mg₂(NH)₃ and Li₄(BH₄)(NH₂)₃. In contrast, the 6-9-12 and 6-9-18 systems exhibit an apparent two-step mechanism: initial Mg(NH₂)₂–LiH reaction at lower temperatures, followed by Mg(NH₂)₂–LiBH₄ reactions at higher temperatures as evidenced by Fig. 5. The persistence of MgNH in the 6-9-12 system and its disappearance in the 6-9-18 system suggests compositionally driven changes in reaction intermediates.

With the 6-9-24 system, the LiBH₄ content dominates the reaction pathway from the outset. The early interaction between Mg(NH₂)₂ and LiBH₄, coupled with minimal LiH involvement, suggests that once LiBH₄ exceeds a critical concentration, it controls the dehydrogenation mechanism. This may explain both the low-temperature release and the unique structural features observed.

The consistent observation of the 1.3° low-angle peak across LiBH₄ rich samples suggests the formation of a metastable superstructure like phase, previously unreported for such Mg(NH₂)₂–LiH–LiBH₄ systems. A previously reported low angle peak for an intermediate in the decomposition of LiBH_4_ by Mosegaard et al. (called Phase I) showed a peak with a d-spacing of 13.50 Å (using λ = 0.92191 Å), which was unidentified^59^. This is different to the peak observed in the LiBH₄ rich samples here, where a low angle peak with a d-spacing of 15.63 Å (λ = 0.35472 Å) is observed. None of the other reflections associated with this peak in Phase I were observed in the samples herein. In addition, the low angle peak observed by Mosegaard et al.^59^ was reported to appear between 473 K and 573 K, whereas in the 6-9-18 system presented here, it is observed to begin from 388 K.

At 500 K in the *x* = 18 and 24 composites, there are broad peaks over the region from ca. 4°–7° 2*θ* indicating either a molten or amorphous phase. For the *x* = 18 composite visual observations from heating in a round-bottom flask up to 480 K indicates that this is likely related to a molten phase. The onset temperature of these either amorphous or molten phases, alongside the metastable phases such as 1.3° 2θ low-angle peak may play a central role in promoting faster hydrogen release while preventing NH_3_ generation. Vajo et al.^60,61^ have shown that the presence of multiple solid phases in a material hinders the kinetics of the solid phase transformations that are key to hydrogen release. These solid-solid reactions can only occur when particles are in physical contact at the atomic scale, limiting the kinetics of the material as transport rates are often slow in solids. When a eutectic electrolyte, such as 0.725LiBH_4_/0.275KBH_4_, is added the transport rates of the various species in the reaction improve as the ions can diffuse through the electrolyte, leading to an increase in the (de)hydrogenation reactions^61^. A potential explanation behind the reduction in the onset temperature for the *x* = 18, 24 systems is that the molten or amorphous character and the 1.3° low-angle peak here are related to an unreported eutectic electrolyte that is being formed in-situ, increasing transport rates and lowering the kinetic barrier of the reaction, with the 6-9-18 system having the most optimal ratio of LiBH₄ for this electrolyte.

The observed primitive cubic cell with a lattice parameter of 3.02 Å, is unreported in previous experiments involving 6Mg(NH₂)₂–9LiH–xLiBH₄ systems. Calcium-nitrogen systems have been investigated for research into NH_3_ synthesis, and a cubic structure for Ca_2_NH have been experimentally verified^62^, however, there has never been a reported experimental observation of a similar Mg_2_NH phase, with a report from 2009 proposing it as a potential compound in complex hydride systems^63^. Initial observations from this data indicate a cubic structure observed in the 6-9-12, 6-9-18 and 6-9-24 samples after 420 K, we propose the cubic cell observed is Mg_2_NH.

This primitive cubic cell, combined with the molten or amorphous character over the borohydride peaks between 4–7° 2*θ* and the superstructure like low angle peak at 1.3° offers interesting insights suggesting a different pathway from the 6-9-1 and 6-9-6 systems. From the data presented in this paper the most likely pathway is evidenced by the changing of Mg phases in the 6-9-18 sample compared to the 6-9-1, assuming the primitive cell is Mg_2_NH:

$${{\rm{Step}}}\;1: \;6{Mg}{\left(N{H}_{2}\right)}_{2}\,+\,9\,{LiH}\,+\,18{LiB}{H}_{4}\rightarrow \,6{MgNH}\,+\,A\, +\,{yLiB}{H}_{4}\,+\,9{H}_{2}$$

Step 2: $$6{MgNH}\,+{yLiB}{H}_{4}+A\,\rightarrow \,3M{g}_{2}{NH}\,+A+B+\,3.5{H}_{2}$$

Where A is likely a superstructure containing Li-B-N-H. Above 420 K the SR-PXRD highlights the MgNH reaction with *y*LiBH_4_ to release hydrogen, forming structure B. B has broad peaks across the region where borohydride peaks lie, indicative of a molten borohydride phase. This is likely to be an ammoniated borohydride Li(NH_3_)_x_(BH_4_). This is plausible as we know from the literature that ammoniated LiBH_4_ can be molten when x = 2 at room temperature, and when x = 1 it is molten at 331 K^64^. In the 6-9-18 and 6-9-24 systems molten phases are observed from 340 K, indicating that any Li(NH_3_)_x_(BH_4_) phase will have an x < 1. Preliminary experiments have indicated that the 6-9-18 sample has a conductance of 4 ×10^−7^ S cm^−1^ at 303 K, assuming the conductivity is dominated by the ammoniated phase, this would mean x < 0.5, possibly as low as 0.2 compared to the literature^64^. If we assume x = 0.2 for an Li(NH_3_)_x_(BH_4_) phase as B then working backwards through the mechanism, allows us to postulate an empirical formula for the large superstructure, A, based on a two step pathway for the 6-9-18 system:

$${{\rm{Step}}}\;1: 6{Mg}{\left(N{H}_{2}\right)}_{2}\,+\,9\,{LiH}\,+\,18{LiB}{H}_{4}\rightarrow \,6{MgNH}\, + \,3\,L{i}_{4}B{N}_{2}{H}_{7}\,+\,15{LiB}{H}_{4}\,+\,9{H}_{2}$$

$${{\rm{Step}}}\;2: 6{MgNH}\,+15{LiB}{H}_{4}+\,3\,L{i}_{4}B{N}_{2}{H}_{7}\,\rightarrow \,3M{g}_{2}{NH}\, +\,3\,L{i}_{4}B{N}_{2}{H}_{7}+15{Li}{\left(N{H}_{3}\right)}_{0.2}(B{H}_{4})+\,3.5{H}_{2}$$

To conclude, we report the so far lowest gas-phase reversible hydrogen release temperatures in these Reactive Hydride Composites, showing the potential of further optimisation of such storage materials for hydrogen storage applications, in the range of low-grade waste heat from a fuel cell. We demonstrate that increasing the LiBH₄ content well beyond the typically explored regime leads to qualitatively different behaviour in Mg(NH₂)₂–LiH–LiBH₄ Reactive Hydride Composites. The 6-9-18 and 6-9-24 compositions exhibit fundamentally different reaction pathways, distinct from all previously reported lower-ratio systems, and these pathways enable the lowest gas-phase reversible hydrogen-release temperatures achieved to date in RHCs. This highlights a breakthrough for off-board and on-board hydrogen storage applications for these materials, being reversible and at temperatures that can utilising low-grade waste heat from a fuel cell.

Most notably, SR-PXRD reveals two intermediate phases, neither of which has been previously identified in RHC chemistry despite two decades of investigation. The evidence of two prior unreported phases, a large superstructure, and a primitive cubic cell represents a step-change in our understanding of how high LiBH₄ environments restructure the amide-hydride reaction landscape. These phases do not appear, and were not predicted, in any lower LiBH₄ systems, underscoring that high borohydride loading drives different chemistry rather than perturbing existing pathways. We propose that this primitive cell is an unreported Mg_2_NH phase, analogous to the Ca_2_NH phase observed^62^.

Crucially, these phases provide previously unseen dehydrogenation routes that (i) dramatically suppress onset temperatures to as low as ~350 K, and (ii) avoid NH₃ release; a dual advance not achieved simultaneously in earlier RHC formulations. The ~100 K downward shift in onset temperature for 6-9-18 compared to the borohydride-free 6-9 composition demonstrates that LiBH₄ does not merely modify kinetics: at these higher concentrations, it functions as a structurally active reagent that defines the entire reaction sequence.

These findings highlight a powerful design principle: increasing LiBH₄ beyond conventional levels does not simply optimise known reactions but unlocks different phases, mechanisms, and performance regimes. Careful compositional tuning enables access to reaction pathways and intermediate phases that promote low-temperature, high-capacity hydrogen storage. This establishes a clear rationale for pursuing high-borohydride RHCs as a distinct subclass of hydrogen-storage materials with properties inaccessible to 6-9–1 or 6-9-6 compositions. Importantly, initial cycling tests performed using a manual Sieverts apparatus confirm that the 6-9-18 composition is reversible, as shown in Supporting Information S6, providing early experimental validation that these pathways can support practical hydrogen uptake and release.

It is worth noting that the conditions these experiments were performed under do not match the transient operating conditions of a PEM fuel cell, and as such, the observed decomposition pathways represent the material’s behaviour under defined laboratory parameters. While the experimental back pressure serves to quantify the fundamental kinetic barriers, real-world fuel cell coupling involves fluctuating pressure and thermal gradients. Therefore, while our results provide the baseline mechanistic understanding required to optimize the system, future work involving integrated fuel cell testing is necessary to validate how these reaction pathways evolve under dynamic operational loading.

To fully exploit these discoveries, future work must examine long-term cycling stability (>50 cycles) and resolve the structures of the observed phases using neutron diffraction, Pair Distribution Function analysis to unravel the amorphous phases, and complementary spectroscopy. Nevertheless, the present results demonstrate that rational compositional tuning can yield reversible hydrogen release below 400 K, bringing RHC materials closer to meeting the 4 wt% target for practical, near-ambient hydrogen-storage applications.

## Methods

### Preparation of 6-9-x samples

All materials were handled under argon atmosphere in an MBraun glovebox with O₂ and H₂O levels below 0.1 ppm. LiH and LiBH₄ (≥95% purity, Merck) were used as received. Mg(NH₂)₂ was synthesised following the method of Cao et al.^40^: MgH₂ was ball-milled using reactive milling^65^ under 7 bar NH₃ for 8 h, followed by heating to 310 °C under static NH₃ (7 bar). The resulting Mg(NH₂)₂ had a purity of >95% (Supporting Information S1).

Reactive hydride composites were prepared with molar ratios of Mg(NH₂)₂:LiH:LiBH₄ of 6:9:*x* (*x* = 1, 6, 12, 18, 24). Each batch comprised 1.2 g of sample ball-milled with twenty-four 9 mm stainless steel balls (316 L grade) in a Fritsch P6 planetary mill using a custom reactive vial^66^. Milling was conducted under 1 bar argon, at 60:1 ball-to-powder ratio, in 2-minute cycles with 1-minute rests for 235 repetitions, alternating direction to limit overheating.

### Thermodynamic measurements

Thermal behaviour was assessed using TGA-DSC-MS (Setaram Setsys 1750 CS Evolution) with a Hiden HPR-20 QMS for gas analysis. Approximately 7 mg of sample was loaded into pierced-lid aluminium crucibles. Complementary DSC measurements were performed on a Netzsch STA 204 HP, using between 1 and 2 mg of sample in ceramic crucibles sealed in aluminium pans (pierced immediately before measurement).

All thermal experiments were performed under flowing high-purity argon (Pureshield, 99.998%, BOC) at 50 mL min⁻¹ for TGA-DSC-MS and 100 mL min⁻¹ for DSC. A heating rate of 2 K min⁻¹ was applied from 298 K to 500 K, chosen to optimise the resolution of sequential reaction events while ensuring consistency across sample measurements.

### Structural characterisation techniques

In-situ synchrotron powder X-ray Diffraction (SR-PXRD) was performed at the ID22 beamline (European Synchrotron Radiation Facility [ESRF], Grenoble), using a 2D PerkinElmer detector and a monochromatic beam (λ = 0.35472752 Å) for fast acquisition and high resolution^67^. Data was collected over 0–30° 2θ with a 0.03° step size. Samples were prepared in 0.7 mm borosilicate capillaries within the ESRF glove box and sealed onto spinners using epoxy glue. Prior testing confirmed no interaction between samples and capillaries (see Supporting Information S2).

2D images were integrated to 1D patterns using Fit2D^68^. Temperature control was achieved via an Oxford Cryostream, with heating rates matching thermal analysis (2 K min⁻¹). Refinements were conducted using FullProf^69^.

## Supplementary information

Supplementary Information

Transparent Peer Review file

## Source data

Source Data

## Data Availability

Data used in the figures is provided as a Source Data File. All TGA, DSC & MS data is available on the University of Nottingham data repository (The data is searchable by the author names), alongside additional XRD data. All data is available on request to the corresponding author. The raw unprocessed SR-PXRD data from ESRF can be found at the following DOIs: 6-9-1 data can be found here: 10.15151/ESRF-DC-2234133904, 6-9-6 data can be found here: 10.15151/ESRF-DC-2234133069, 6-9-12 data can be found here: 10.15151/ESRF-DC-2234115947, 6-9-18 data can be found here: 10.15151/ESRF-DC-2234130698, 6-9-24 data can be found here: 10.15151/ESRF-DC-2234134335. Source data are provided with this paper.
