# Supplementary material for: Reversible hydrogen storage in reactive hydride composites under 400 K
Source: Nat Commun. 2026 Jul 10;17:8533. doi: 10.1038/s41467-026-75313-0 (PMC13482644; doi:10.1038/s41467-026-75313-0)
Supplement: Supplementary file 1 [file 41467_2026_75313_MOESM1_ESM.pdf]

Supplementary information
Reversible hydrogen storage in reactive hydride composites under 400K

Nicholas J. Hall [1], David M. Grant [1], Jacob L. Prosser [1], Jamie E. Ramshaw [1], Muhammad Saad Salman[1], Luke Woodliffe [1], Sweta Munshi [1], Guillaume Esser [2], Sanliang Ling [1], Marek Polanski [3], Yaroslav Filinchuk [2], Hujun Cao [4], Ping Chen [4], Martin Dornheim [1]*

[1] – Advanced Materials Research Group, Faculty of Engineering, University of Nottingham, United Kingdom
[2] – Institute of Condensed Matter and Nanosciences, Université catholique de Louvain, Louvain-la-Neuve, Belgium
[3] – Department of Functional Materials and Hydrogen Technology, Military University of Technology, Warsaw, Poland
[4] – Dalian Institute of Chemical Physics, Chinese Academy of Sciences, China
** – Corresponding author.* david.grant@nottingham.ac.uk

Table of Contents

S1. $Mg(NH_2)_2$ Synthesis Validation 2
S2. Capillary Compatibility Testing 3
S3. Milling Protocol 3
S4. Enthalpy Calculation from DSC 4
S5. TGA calculations of capacity 4
S6. Preliminary 6-9-18 data to demonstrate reversibility 5
S7. Primitive Cubic Phase observed in ESRF measurements 9
S8. Dehydrogenation products on heating to 723 K for the 6-9-12 10

S1. $Mg(NH_2)_2$ Synthesis Validation

$Mg(NH_2)_2$ was synthesised following the protocol of Cao et al. [40]. Briefly, MgH_2 was ball-milled under 7 bar NH_3 for 8 hours, followed by static heating at 310 °C under 7 bar NH_3 for 4 hours. A minimum ratio of 2:1 NH_3 to MgH_2 was maintained throughout the process. Purity was confirmed in Nottingham via powder X-ray diffraction (PXRD), using a Bruker D8 Advance diffractometer with Cu Kα radiation, and matched to reference patterns for $Mg(NH_2)_2$. The overall purity was estimated at >95%, with no crystalline MgO or MgH_2 phases visible (refinement SI Figure 1: Bragg R-factor = 9.12, Rf = 5.54).

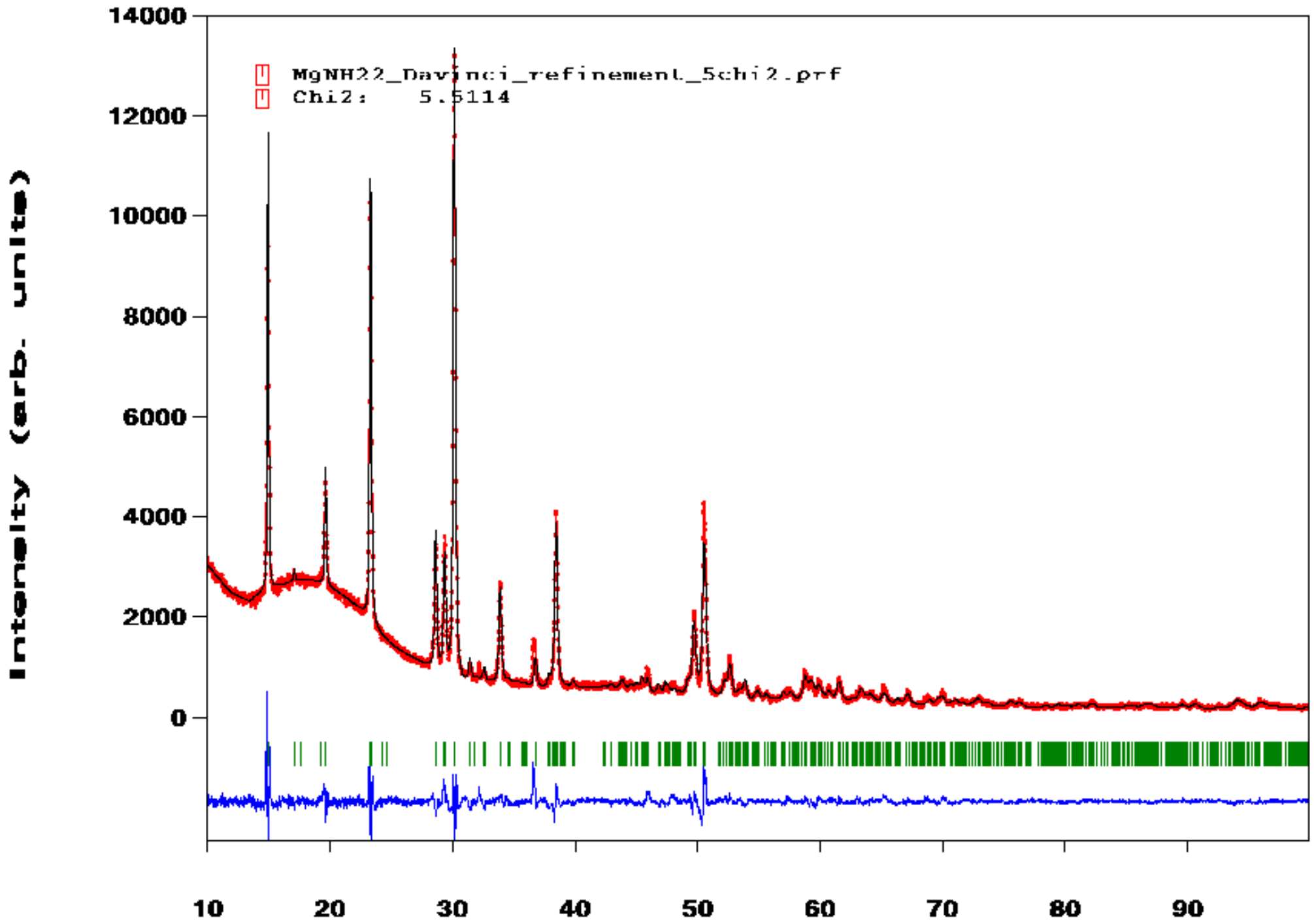

SI Figure 1: Refinement on $Mg(NH_2)_2$ as received using FullProf. Red points are the measured pattern, the black line is the calculated pattern and the blue line is the difference. Green marks are reflection points. XRD was run on a Bruker D8 Advance using a Cu source X-ray tube with a wavelength of 1.54 Å, the background hump between 12-25 2θ is due to amorphous tape protecting the sample from exposure to air

S2. Capillary Compatibility Testing

Prior to synchrotron experiments, control studies were conducted at UCLouvain to verify capillary-material compatibility. RHC samples of $Mg(NH_2)_2$, LiH, $LiBH_4$ were sealed in both borosilicate and sapphire capillaries and subjected to thermal cycling between 298 K and 500 K. No reactions or phase changes attributable to capillary interaction were observed by in situ PXRD at UCLouvain or visual inspection, the only differences related to the crystalline peaks of sapphire that weren't fully masked.

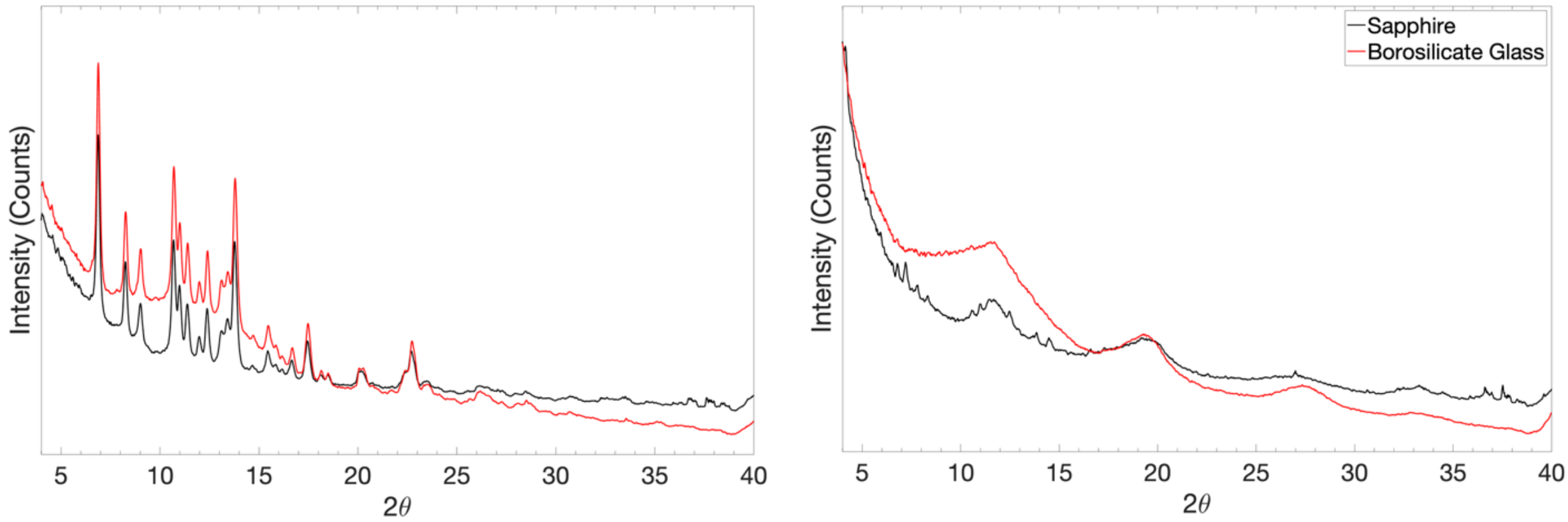

SI Figure 2: Capillary compatibility testing at UCLouvain, using a wavelength of 0.71 Å. (Left) Sapphire and borosilicate glass capillaries with sample inside at RT, (Right) Sapphire and borosilicate glass at 473 K. The differences are due to incomplete masking of the sapphire reflections.

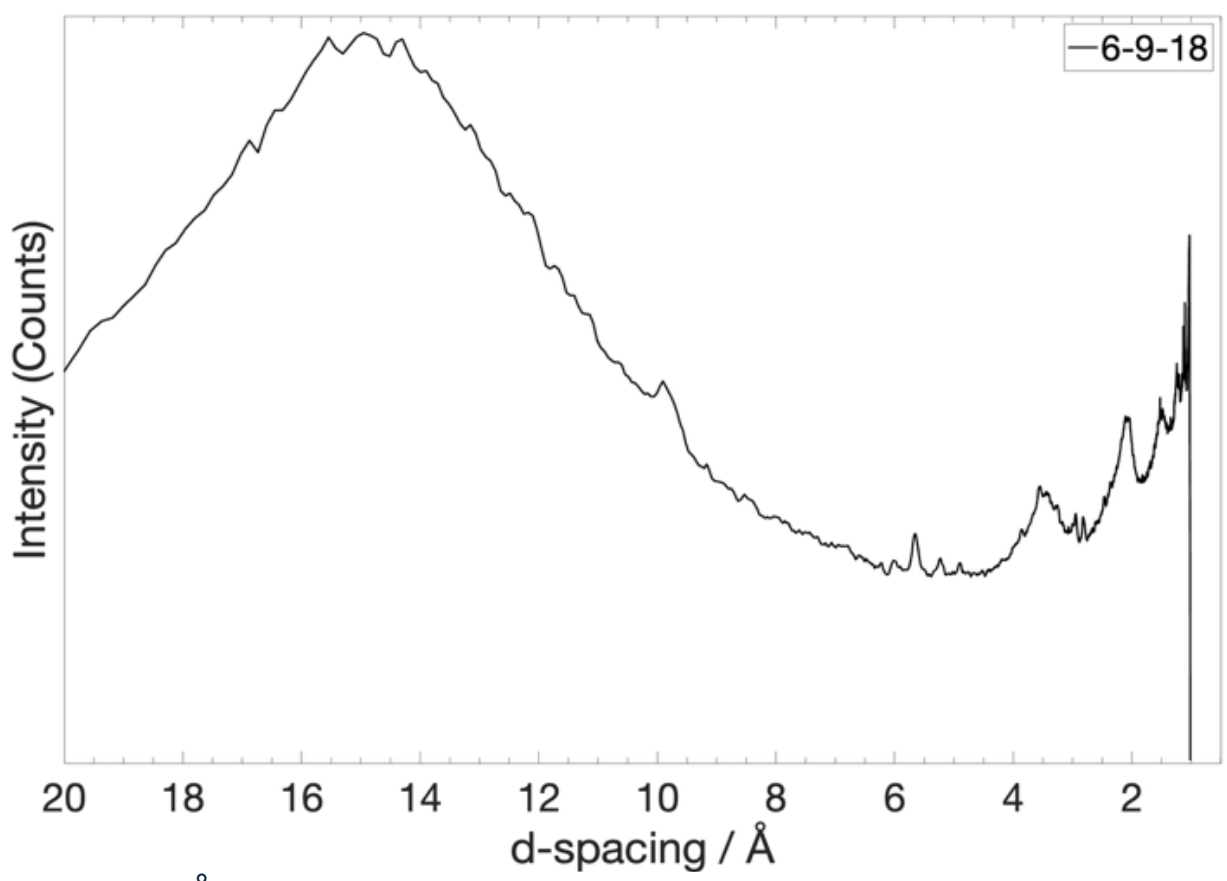

SI Figure 3: Low angle peak at ca. 16 Å , in a sapphire capillary for the 6-9-18 composite, indicating that the observed low angle peak in the SR-PXRD is a genuine artifact and not a processing or capillary issue.

S3. Milling Protocol

The ball-milling procedure was optimised from prior literature on such systems [20] to minimise heat generation and gas loss. Milling was performed in 2-minute intervals with 1-minute rest periods, alternating rotation direction after each cycle. The reactive vial provided by Marek Polanski was selected for its enhanced thermal tolerance and

sealing integrity, reducing the risk of hydrogen loss during mechanical activation. The vial volume was approximately 99 cm^3.

S4. Enthalpy Calculation from DSC

DSC data was baseline corrected and integrated using Netzsch Proteus software. Enthalpy values were normalised to sample mass. The $LiBH_4$ octahedral-to-hexagonal phase transition was used as an internal reference, with a literature value of 275 J g^{-1} for comparison [52]. Experimental deviations from this benchmark were used to infer overlapping reactions and additional thermal events.

S5. TGA calculations of capacity

Uncertainties calculated as 95% confidence interval for systems with 3 or more runs, for less than 3 runs half the range is used as the uncertainty. 6-9-1 and 6-9-6 samples were from the same batches, 6-9-12,6-9-18 and 6-9-24 were across multiple batches.

SI Table 1: TGA runs for the 6-9-1 system

Date	Ramp rate (K per min)	Temperature wt% taken at (Furnace temperature in C)	Wt %
28/11/24	10	250	3.28
20/02/25	2	250	4.72
**Average**	**-**	**-**	**4.00 ± 0.72**

SI Table 2: TGA data for the 6-9-6 System

Date	Ramp rate (K per min)	Temperature wt% taken at (Furnace temperature in C)	Wt %
02/12/24	10	250	3.86
20/02/25	2	250	4.94
**Average**	**-**	**-**	**4.40 ± 0.54**

SI Table 3: TGA data for the 6-9-12 System

Date	Ramp rate (K per min)	Temperature wt% taken at (Furnace temperature in C)	Wt %
11/11/24	10	250	2.45
12/11/24	10	250	2.57
06/01/25	2	250	2.51
27/02/25	2	250	3.09
**Average**	**-**	**-**	**2.66 ± 0.47**

SI Table 4: TGA data for the 6-9-18 System

Date	Ramp rate (K per min)	Temperature wt% taken at (Furnace temperature in C)	Wt %
27/11/24	10	250	3.52
28/11/24	10	250	3.05
18/12/24	2	250	3.12
10/03/25	2	250	3.05
19/03/25	2	250	2.84
**Average**	**-**	**-**	**3.12 ± 0.31**

SI Table 5: TGA data for the 6-9-24 System

Date	Ramp rate (K per min)	Temperature wt% taken at (Furnace temperature in C)	Wt %
04/12/24	10	250	3.01
09/01/25	2	250	2.86
20/02/25	2	250	3.03
**Average**	**-**	**-**	**2.97 ± 0.23**

S6. Preliminary 6-9-18 data to demonstrate reversibility

This is data taken from some testing conducted with the 6-9-18 sample on an in-house manual sieverts apparatus in Nottingham. The sample was dehydrogenated under dynamic vacuum at 453 C for an hour to ensure it had been dehydrogenated. We then conducted numerous tests on the sample, holding at different temperatures using up to 100 bar H_2. Values obtained for the gravimetric capacity were in line with the TG measurements. SI Figure 3 is an example of the hydrogenation conducted at 433 K, obtaining just over 3 wt%:

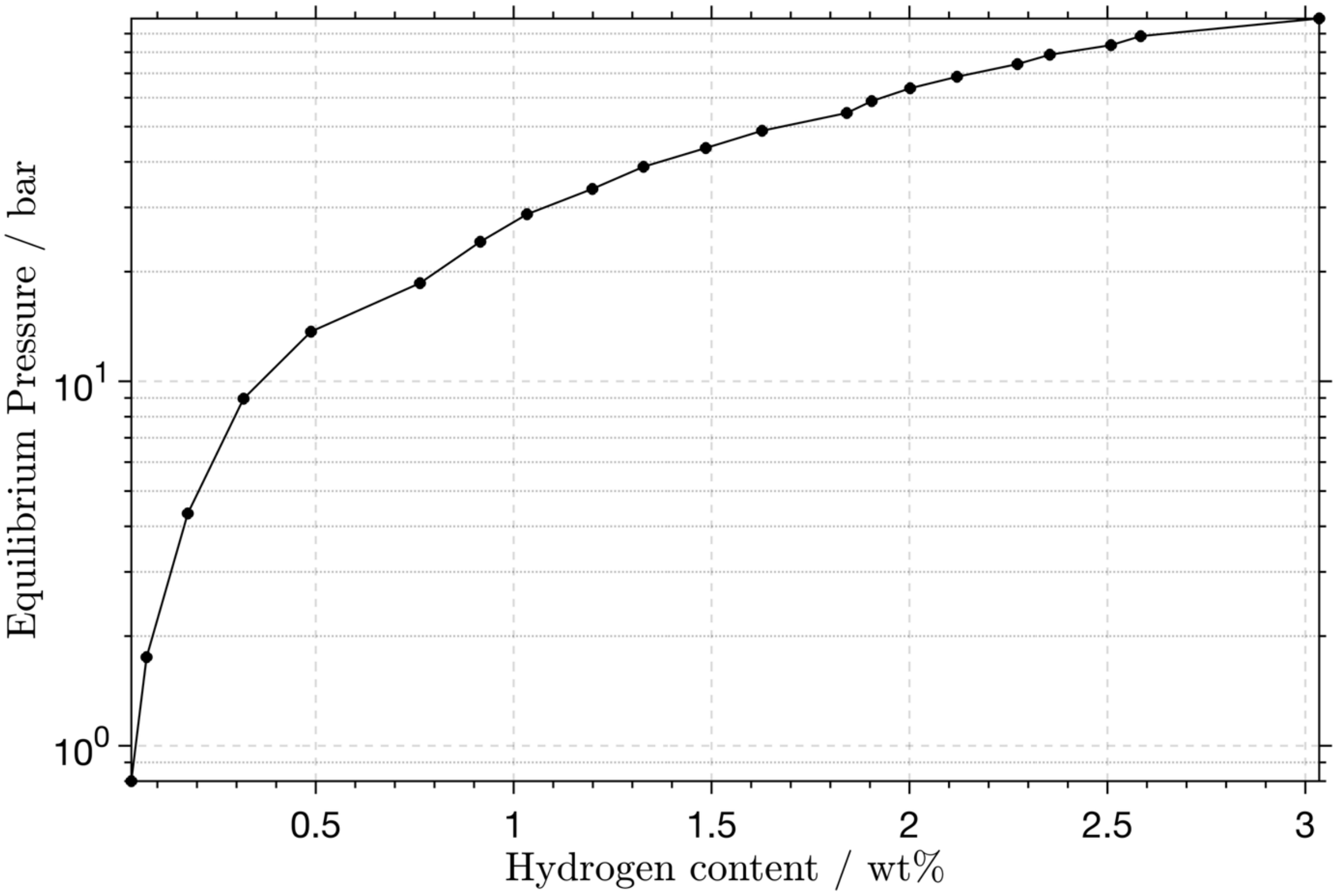

SI Figure 3: Hydrogenation PCI conducted at 433 K

An example of a dehydrogenation at 403 K is also displayed here (SI Figure 4). The measured gravimetric capacity here is higher than the 3.12 wt% seen in the TG experiments:

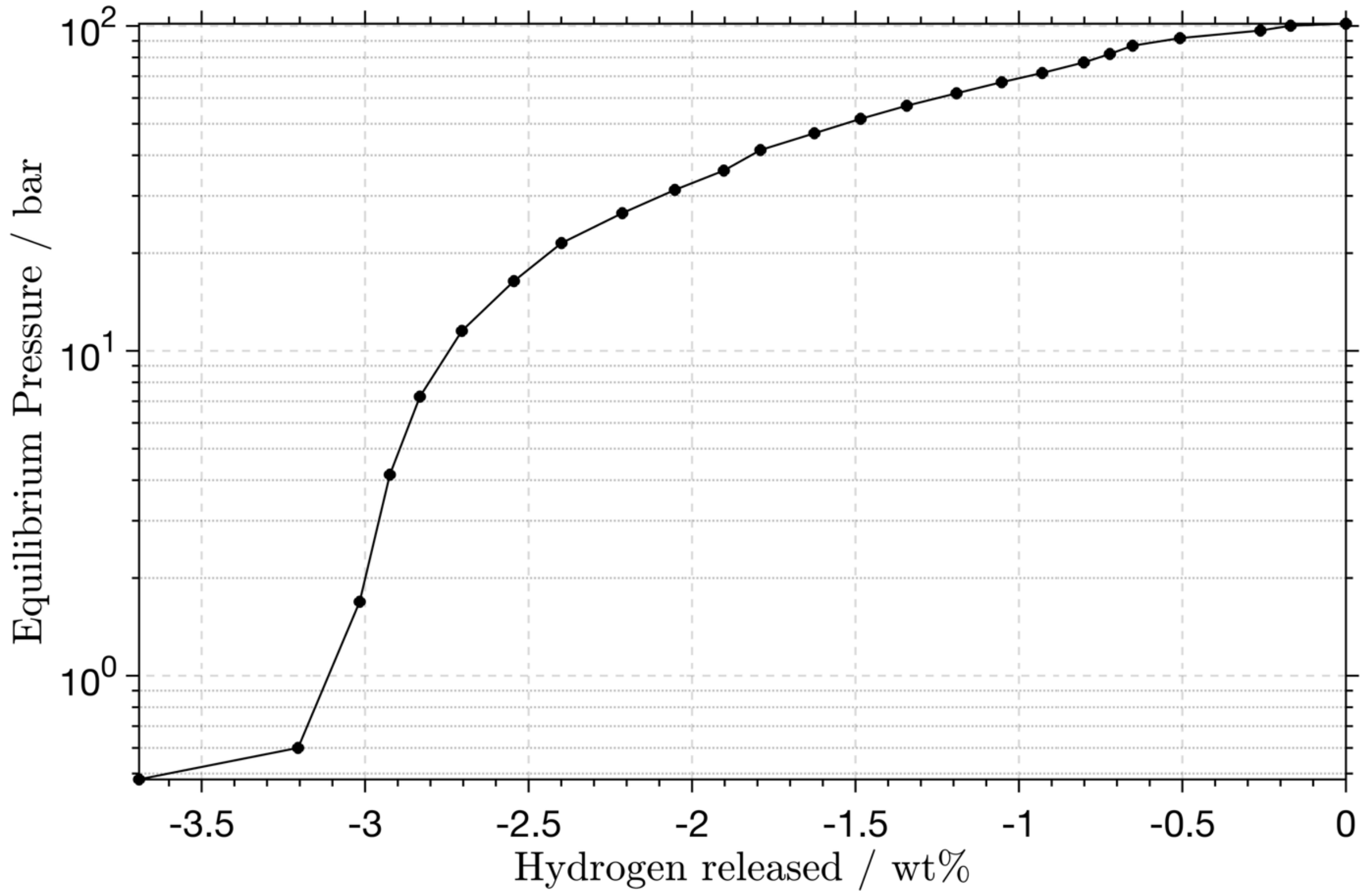

SI Figure 4: Dehydrogenation PCI conducted at 403 K

A TGA-DSC-MS run was conducted on the 6-9-18 sample following 3 cycles to assess reversibility. Due to significantly slower kinetics compared to the other samples, a reduced heating rate of 1 K min^{-1} was employed rather than the standard 2 K min^{-1}, ensuring capturing the dehydrogenation of the reversible fraction up to 500 K. Whilst still achieving 3 wt%, the cycled sample exhibits notably different dehydrogenation behaviour; the low-temperature release observed in the fresh sample is absent, with hydrogen release commencing at approximately 390 K. After cycling two distinct, well-separated endothermic events (one $LiBH_4$ orthogonal to hexagonal phase transition and the other unknown) are observed around the H_2 release point. The origin of this behaviour warrants further investigation and will be addressed in future work. This is shown in SI Figure 5.

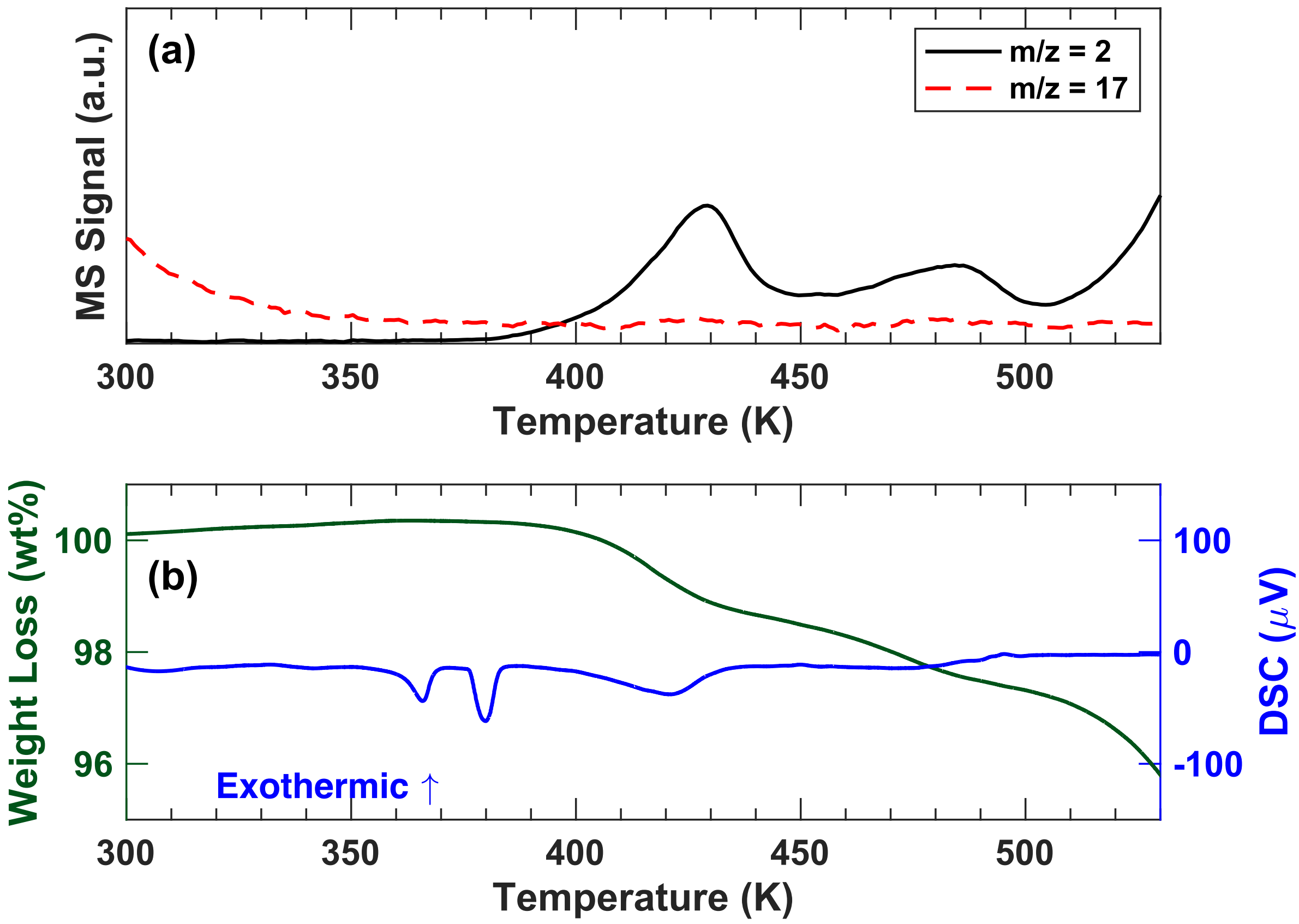

SI Figure 5: TGA-DSC-MS of a 6-9-18 sample cycled 3x above 400 K, cooled to RT and then run. (a) highlights the MS signal, in arbitrary units, with (b) illustrating the TG-DSC data.

S7. Primitive Cubic Phase observed in ESRF measurements

As mentioned in the manuscript, this is the primitive cell observed at 500 K in the 6-9-18 sample (SI Figure 5). The reflection pattern is weak, but noticeable in the sample, indicating that this phase is possibly soluble in the melt phases present.

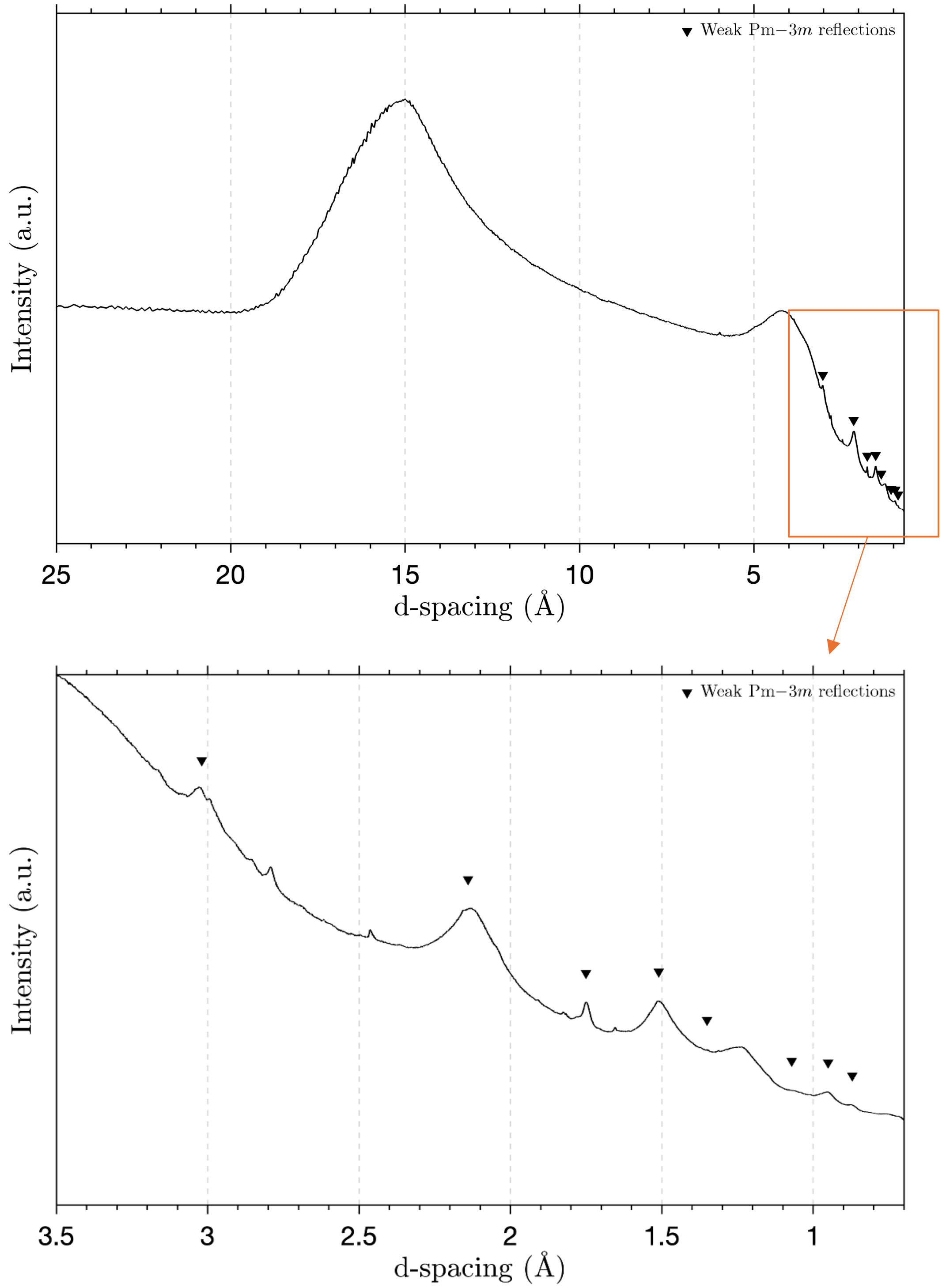

SI Figure 6: Primitive cubic cell observed at 500 K in the 6-9-18 sample. (Top) Full scan in d-spacing, (Bottom) Closer zoom in on peaks identified as Pm-3m cell

S8. Dehydrogenation products on heating to 723 K for the 6-9-12

A sample of the 6-9-12 was heated up on a manual sieverts under dynamic vacuum to 723 K. It was held for 30 minutes before being slowly cooled to room temperature. The resulting material was tested via PXRD on a Bruker DaVinci D8 Advance, indicating the presence of stable materials such as Mg_3N_2.

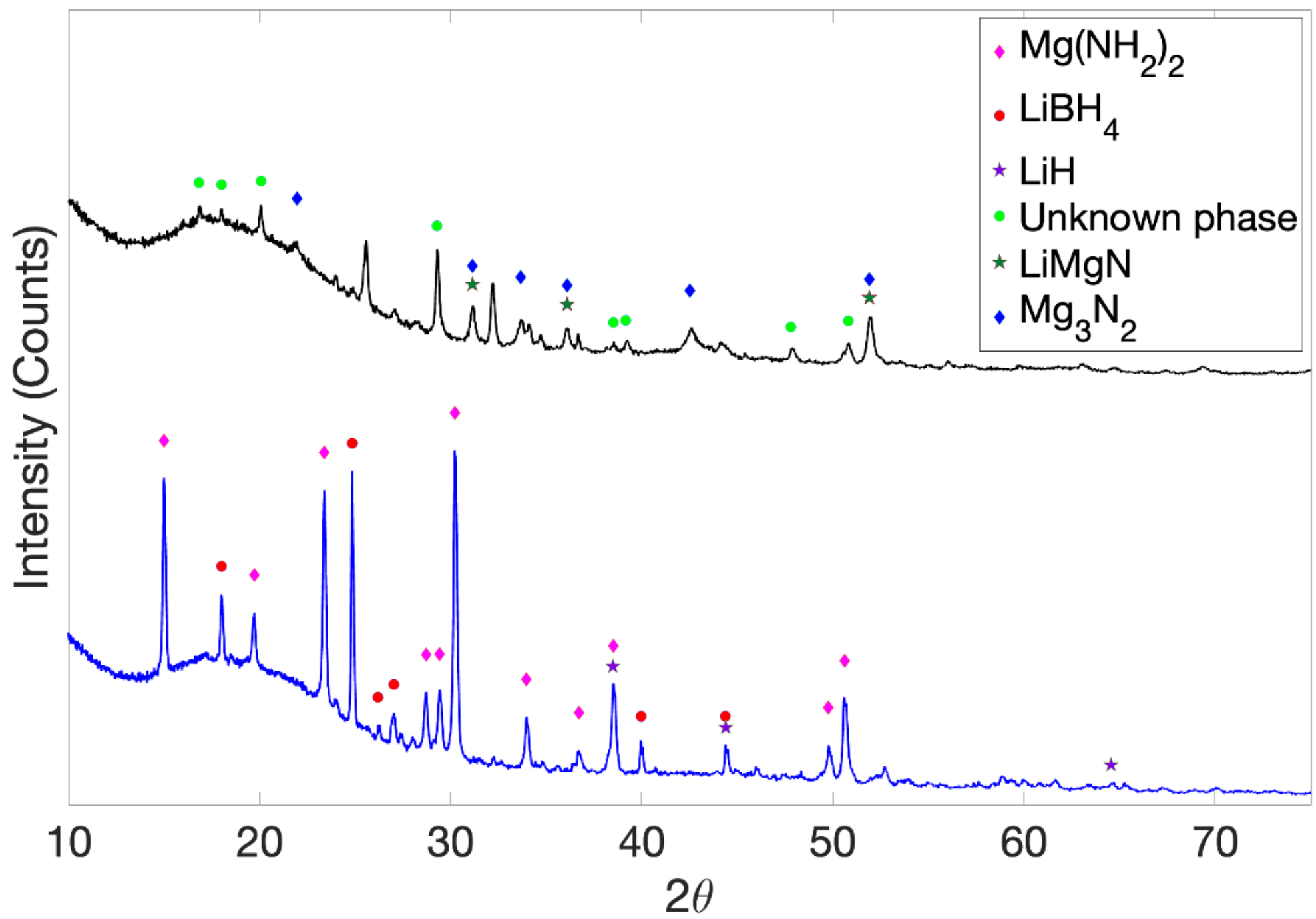

SI Figure 7: 6-9-12 before dehydrogenation (blue) and after heating to 723 K to dehydrogenate (black). Peaks were indexed using Fullprof. XRD was run on a Bruker D8 Advance using a Cu source X-ray tube with a wavelength of 1.54 Å, the background hump between 12-25 2θ is due to amorphous tape protecting the sample from exposure to air
