# Supplementary material for: Reversible hydrogen storage in reactive hydride composites under 400 K
Source: Nat Commun. 2026 Jul 10;17:8533. doi: 10.1038/s41467-026-75313-0 (PMC13482644; doi:10.1038/s41467-026-75313-0)
Supplement: Supplementary file 2 [file 41467_2026_75313_MOESM2_ESM.pdf]

nature portfolio

Peer Review File

Reversible hydrogen storage in reactive hydride composites under 400 K

Corresponding Author: Professor David Grant

This file contains all reviewer reports in order by version, followed by all author rebuttals in order by version.

Version 1:

Reviewer comments:

Reviewer #1

(Remarks to the Author)
The manuscript authored by Nicholas Hall and co-workers investigates the hydrogen storage properties of reactive hydride composites (RHCs) based on $Mg(NH_2)_2$, LiH, and $LiBH_4$. In particular, the study focuses on the impact of $LiBH_4$ content on the operating conditions of the composite system. The authors found that when unusually high amounts of $LiBH_4$ are employed, the dehydrogenation temperature decreases to below 100 °C. This finding is relevant to the hydrogen storage community, as it demonstrates the tangible potential to achieve substantial hydrogen storage capacity under conditions compatible with PEM fuel cell operation. The work supports the conclusions and claims, and the utilized methodology is sound. The manuscript is therefore of interest to the audience of this journal and should be considered for publication after addressing the following points:
· The authors claim, "Solid-state hydrogen storage offers an appealing alternative by increasing volumetric energy density beyond that of cryogenic and compressed gas approaches without the need for extreme pressures or cryo-temperatures"; however, the volumetric advantage of solid-state storage over cryogenic storage is purely at the material storage level. To the best of my knowledge, the so far reported solid-state storage systems have a volumetric hydrogen density lower than those of cryogenic systems. Please comment or correct the text.
· The authors claim, "The reversibility of $LiBH_4$ is equally challenging, needing 873 K and 350 bar of hydrogen for rehydriding." Please mention that this is just a partial reversibility due to the segregation of boron in stable compounds, e.g., boron $Li_2B_{12}H_{12}$, and its loss via the formation of volatile B-containing species such as B_2H_6.
· The authors claim, "The RHC approach trades some theoretical hydrogen capacity for improved kinetics, system cyclability, and practical viability through operating temperatures closer to the operating temperature of a fuel cell." So far, operating temperatures closer to the operating temperature of a fuel cell have been just theoretically predicted and not achieved.
· Please correct this: "new complex structures and reversible pathways.."
· The author states, "These later events likely involve the decomposition of excess $LiBH_4$ and formation of thermodynamically stable phases such as Mg_3N_2 (SI Figure 6)." Is it known what $LiBH_4$ is forming after decomposition?
· The authors should comment on the fact that the experimental conditions used for the various decomposition measurements—particularly the applied hydrogen back pressure—do not closely match the operating conditions of a practical hydrogen storage system coupled with a PEM fuel cell. This discrepancy could significantly influence the material's behavior under real operating scenarios, affecting both the reaction pathway and the observed operating temperatures. It would therefore be useful if the authors discussed how these experimental conditions might influence the extrapolation of their results to realistic hydrogen storage applications.

Reviewer #2

(Remarks to the Author)
This work presents a reactive hydrogenation composite of 6Mg(NH2)2-9LiH-xLiBH4 system, in which 6-9 with high LiBH4 amount can yield reversible hydrogen release below 400 K, bringing RHC closer to meeting the 4 wt% target for practical, near-ambient hydrogen-storage applications. Although the 6-9 and 6-9-1 systems were discovered more than a decade ago, the reversible hydrogen storage performance with high capacity at low temperatures brought by the addition of high-capacity LiBH4 is a very meaningful discovery in the community recently. Its reversible hydrogen storage mechanism is also worthy of further exploration. However, there are still some questions and suggestions regarding this manuscript, the reviewer looks forward to the author's addresses before recommending acceptance.

1. The author reported the dehydrogenation process of the 6-9- LBH materials system, and the intermediate phases have also been characterized and elucidated. However, the study on the reversible hydrogen absorption process of the sample is relatively cursory. The paper only mentions reversible hydrogen storage performance in the Supplementary Information (SI), but does not mention the intermediate and final product phases. Corresponding characterization experiments are also relatively lacking.
2. The 6-9-18 and 6-9-24 systems can reversible de/re-hydrogenation. However, the reversibility of those initial/intermediate phases are unclear. For example, the MgNH identified in 6-9-12 system according to the in-situ XRD pattern of Figure 6. It seems to be stable only below 450K. If the de/re-hydrogenation were conducted at the temperature below 450K, how about the stability of MgNH? Is it reversible?
3. For 6-9-18 sample in SI Figure 4, more P-I isotherms de/re-hydrogenation curves at different temperature are strongly recommended within the temperature range of 350K to 500K. The equilibrium plateaus and de/re-hydrogenation enthalpy should be precisely identified.
4. The peak temperatures of MS and DSC in Figure 1 should be marked.
5. Why is there no endothermic peak of phase transition for LiBH4 in Figure 3, 6-9-1? The text should provide an appropriate explanation.

Reviewer #3

(Remarks to the Author)

The comments for authors are given below.

1.As shown in Fig.2, in the TG analysis, the hydrogen desorption starting from ~350K of Sample 6-9-18 is lower than that of Sample 6-9-24, but in the MS analysis, the weight loss of Sample 6-9-18 is larger than that of Sample 6-9-24. This contradiction implies an incorrect data or a possible emission of NH3. Furthermore, the TG results showed that the weight losses starting from ~350K for the samples 6-9-18 and 6-9-24 are 1.2 wt.% and 1 wt.%, respectively, which corresponding to about 38.5% and 33.7% of the total released hydrogen, respectively. The weight losses in MS result contradict the very small peaks in TG result. The results should be carefully checked again and corrected.

2.The cyclability at low temperature was not tested in the paper. It is highly suggested to measure the rehydrogenated sample by TGA-MS to confirm the reversibility of the release starting from ~350K.

Version 2:

Reviewer comments:

Reviewer #1

(Remarks to the Author)
All requested changes and modifications have been implemented. The responses to the raised points are also satisfactory. The manuscript is suitable for acceptance in its current form.

Reviewer #2

(Remarks to the Author)
All concerns from the reviewer have been well addressed, I recommend that the manuscript can be accepted as is.

Reviewer #3

(Remarks to the Author)
The authors have revised the manuscript in accordance with the reviewer's comments. I recommend accepting this manuscript for publication after minor revisions.
1.Based on the results presented in SI Figure 5, reversible hydrogen release initiates at approximately 390 K. We therefore recommend that the sentence "achieving reversible hydrogen release at temperatures starting as low as 350 K (77 ˚C)" in the abstract be revised accordingly.
2.The heating rate of 1 K min^{-1} should be added to the sentence containing "…with H_2 onset shifting to 388 K" on line 9 of page 13.

Open Access This Peer Review File is licensed under a Creative Commons Attribution 4.0 International License, which permits use, sharing, adaptation, distribution and reproduction in any medium or format, as long as you give appropriate credit to the original author(s) and the source, provide a link to the Creative Commons license, and indicate if changes were made.
In cases where reviewers are anonymous, credit should be given to 'Anonymous Referee' and the source.
The images or other third party material in this Peer Review File are included in the article's Creative Commons license, unless indicated otherwise in a credit line to the material. If material is not included in the article's Creative Commons license and your intended use is not permitted by statutory regulation or exceeds the permitted use, you will need to obtain permission directly from the copyright holder.
To view a copy of this license, visit https://creativecommons.org/licenses/by/4.0/

Response to reviewers

Location of changes listed in **bold.** The changes are in yellow and are both here and on the paper manuscript NCOMMS-25-96252A-Z and its Supplementary Information. Thank you.

Reviewer 1

· The authors claim, “Solid-state hydrogen storage offers an appealing alternative by increasing volumetric energy density beyond that of cryogenic and compressed gas approaches without the need for extreme pressures or cryo-temperatures”; however, the volumetric advantage of solid-state storage over cryogenic storage is purely at the material storage level. To the best of my knowledge, the so far reported solid-state storage systems have a volumetric hydrogen density lower than those of cryogenic systems. Please comment or correct the text.

See page 1, 6 lines from bottom of page

At the material level solid-state hydrogen storage offers....

· The authors claim, “The reversibility of $LiBH_4$ is equally challenging, needing 873 K and 350 bar of hydrogen for rehydriding.” Please mention that this is just a partial reversibility due to the segregation of boron in stable compounds, e.g., boron $Li_2B_{12}H_{12}$, and its loss via the formation of volatile B-containing species such as B_2H_6.

See page 2, 20 lines from bottom

Full reversibility is hindered by two primary mechanisms: the segregation of boron into stable compounds (such as $Li_2B_{12}H_{12}$) and its subsequent loss through volatile species like diborane (B_2H_6).

· The authors claim, “The RHC approach trades some theoretical hydrogen capacity for improved kinetics, system cyclability, and practical viability through operating temperatures closer to the operating temperature of a fuel cell.” So far, operating temperatures closer to the operating temperature of a fuel cell have been just theoretically predicted and not achieved.

See page2 , 6 lines from bottom

... practical viability through theoretical operating temperatures closer to the operating temperature ...

· Please correct this: “new complex structures and reversible pathways..”

See page 4 , 9 lines from bottom

... with high ratios of $LiBH_4$ which form new complex structures through reversible pathways.

· The author states, “These later events likely involve the decomposition of excess $LiBH_4$ and formation of thermodynamically stable phases such as Mg_3N_2 (SI Figure 6).” Is it known what $LiBH_4$ is forming after decomposition?

See page 6, 8 lines from top

... and formation of thermodynamically stable phases such as Mg_3N_2 and LiMgN (SI Figure 6), as well as probable B_2H_6 release.

· The authors should comment on the fact that the experimental conditions used for the various decomposition measurements—particularly the applied hydrogen back pressure—do not closely match the operating conditions of a practical hydrogen

storage system coupled with a PEM fuel cell. This discrepancy could significantly influence the material's behavior under real operating scenarios, affecting both the reaction pathway and the observed operating temperatures. It would therefore be useful if the authors discussed how these experimental conditions might influence the extrapolation of their results to realistic hydrogen storage applications.

See page 16, paragraph at top of page

It is worth noting that the conditions these experiments were performed under do not match the transient operating conditions of a PEM fuel cell, and as such, the observed decomposition pathways represent the material's behaviour under defined laboratory parameters. While the experimental back pressure serves to quantify the fundamental kinetic barriers, real-world fuel cell coupling involves fluctuating pressure and thermal gradients. Therefore, while our results provide the baseline mechanistic understanding required to optimize the system, future work involving integrated fuel cell testing is necessary to validate how these reaction pathways evolve under dynamic operational loading.

Reviewer 2

1. The author reported the dehydrogenation process of the 6-9- LBH materials system, and the intermediate phases have also been characterized and elucidated. However, the study on the reversible hydrogen absorption process of the sample is relatively cursory. The paper only mentions reversible hydrogen storage performance in the Supplementary Information (SI), but does not mention the intermediate and final product phases. Corresponding characterization experiments are also relatively lacking.

See page 12, lines 5,6 and 7 from top

The thermal and structural analysis presented here reveals insights into the first cycle dehydrogenation of $Mg(NH_2)_2$–LiH–$LiBH_4$ RHCs and their further optimisation potential. In addition, to verify reversibility PCI measurements and thermal characterisation was conducted over 3 cycles (SI S6).

2. The 6-9-18 and 6-9-24 systems can reversible de/re-hydrogenation. However, the reversibility of those initial/intermediate phases are unclear. For example, the MgNH identified in 6-9-12 system according to the in-situ XRD pattern of Figure 6. It seems to be stable only below 450K. If the de/re-hydrogenation were conducted at the temperature below 450K, how about the stability of MgNH? Is it reversible?

With respect, MgNH is reversible and has been shown in literature. We have added a sentence to make this clear

See page 9, 5 lines from top

MgNH formation, a stable intermediate previously found in the decomposition of $Mg(NH_2)_2$ and in prior 6-9-12 characterisation[17,24], began at 440 K ...

3. For 6-9-18 sample in SI Figure 4, more P-I isotherms de/re-hydrogenation curves at different temperature are strongly recommended within the temperature range of 350K to 500K. The equilibrium plateaus and de/re-hydrogenation enthalpy should be precisely identified.

For this system as can be seen in SI there is no equilibrium plateau . This was not in the remit of the paper and exploring enthalpy using calorimetry and high pressure PCIs at multiple temperature would form future work.

4. The peak temperatures of MS and DSC in Figure 1 should be marked.

See Figure 1

5. Why is there no endothermic peak of phase transition for LiBH4 in Figure 3, 6-9-1? The text should provide an appropriate explanation.

See paragraph added on Page 8

... except 6-9-1 displayed a first endothermic event near 380 K, typically assigned to the $LiBH_4$ orthorhombic to hexagonal phase transition. The 6-9-1 in the measurements here is in line with prior experiments by Gizer et al, where the phase transition is unobserved [25], and this is unexplained.

Reviewer 3

1.As shown in Fig.2, in the TG analysis, the hydrogen desorption starting from ~350K of Sample 6-9-18 is lower than that of Sample 6-9-24, but in the MS analysis, the weight loss of Sample 6-9-18 is larger than that of Sample 6-9-24. This contradiction implies an incorrect data or a possible emission of NH3. Furthermore, the TG results showed that the weight losses starting from ~350K for the samples 6-9-18 and 6-9-24 are 1.2 wt.% and 1 wt.%, respectively, which corresponding to about 38.5% and 33.7% of the total released hydrogen, respectively. The weight losses in MS result contradict the very small peaks in TG result. The results should be carefully checked again and corrected.

See Figure 2 caption. The reviewer got a bit confused on this based on the MS hydrogen signal is in arbitrary units which are not normalised as different sample masses were used. So we have added text into the caption in figure 2 to clarify this and to avoid similar confusion from the reader.

*Figure 1: (Left) TGA-MS data, highlighting the difference in reaction of the 6-9-18 and 6-9-24 samples against the previously studied 6-9-1,6-9-6 and 6-9-12 systems. The H_2 signal from the MS (m/z = 2) has arbitrary height due to differing sample masses on the TGA (Right) A graphic visualisation of the TGA-MS data illustrating gravimetric capacities (TG), detectable H_2 onset temperatures (MS), major release point and temperature range. Major H_2 release is calculated from the derivative of the TG data, highlighting the area of steepest weight loss. * Indicates kinetic challenges in these systems from the literature*

2.The cyclability at low temperature was not tested in the paper. It is highly suggested to measure the rehydrogenated sample by TGA-MS to confirm the reversibility of the release starting from ~350K.

We have done this and added figure 5 in SI plus a paragraph of explanation on page 7 in the SI

A TGA-DSC-MS run was conducted on the 6-9-18 sample following 3 cycles to assess reversibility. Due to significantly slower kinetics compared to the other samples, a reduced heating rate of 1 K min^{-1} was employed rather than the standard 2 K min^{-1}, ensuring capturing the dehydrogenation of the reversible fraction up to 500 K. Whilst still achieving 3 wt%, the cycled sample exhibits notably different dehydrogenation behaviour; the low-temperature release observed in the fresh sample is absent, with hydrogen release commencing at approximately 390 K. After cycling two distinct, well-separated endothermic events (one $LiBH_4$ orthogonal to hexagonal phase transition and the other unknown) are observed around the H_2 release point. The origin of this

behaviour warrants further investigation and will be addressed in future work. This is shown in SI Figure 5
